# The burden of COVID-19-related intensive care admissions in the Nordic countries, 2020–2023

**DOI:** 10.1186/s12879-026-13528-8

**Published:** 2026-05-19

**Authors:** Elina Seppälä, Niels Bindslev, Sophie Gubbels, Karina Lauenborg Møller, Simopekka Vänskä, Tryggvi Hjörtur Oddson, Arna Harðardóttir, Maríanna Þórðardóttir, Lisa Mather, Marie Jansson Mörk, Moa Rehn, Knut Lönnroth, Eirik Alnes Buanes, Preben Aavitsland

**Affiliations:** 1 Norwegian Institute of Public Health, Oslo, Norway; 2 Statens Serum Institut, Copenhagen, Denmark; 3 Finnish Institute for Health and Welfare, Helsinki, Finland; 4 Directorate of Health, Reykjavik, Iceland; 5 Public Health Agency of Sweden, Stockholm, Sweden; 6 Government of Åland, Mariehamn, Finland; 7 Department of Global Public Health, Karolinska Institutet, Stockholm, Sweden; 8 Norwegian Intensive Care and Crisis Registry, Bergen, Norway; 9 Pandemic Centre, University of Bergen, Bergen, Norway

**Keywords:** COVID-19, Pandemic, Intensive care admission, Nordic countries

## Abstract

**Background:**

The COVID-19 pandemic caused a significant burden on populations and intensive care units (ICU) globally. We assessed the population burden of admissions to the ICU with COVID-19 in the Nordic countries in 2020–2023 to inform pandemic planning.

**Methods:**

We used data from national health registries and applied national case definitions to identify patients admitted to the ICU with and due to confirmed COVID-19. We calculated cumulative incidence per 100,000 inhabitants by country, and described the characteristics of the patients including age, sex, and disease severity.

**Results:**

In 2020–2023, 21,587 patients were admitted to the ICU with COVID-19, of whom > 13,000 patients were admitted due to COVID-19. Sweden had the highest cumulative incidence of patients admitted due to COVID-19 (78.5 per 100,000, *n* = 8,179), and a peak incidence twice that of the other countries. The median age of ICU patients increased over time in all countries, and the majority (62–72%) of patients were men. The longest length of stay in 2020–2021 was recorded in Norway (median 14, lower - upper quartile 7.0–23.5 days), and it decreased to 2, 3 or 4 days in most countries in 2022–2023. Seventy-nine percent of patients received ventilatory support and 25% of patients died, with increasing in-ICU mortality in Norway and Sweden towards 2023. Differences in data sources and case definitions limited the comparability of data from the countries.

**Conclusions:**

The burden of ICU admissions with COVID-19 varied in the Nordic countries and was associated with non-modifiable factors like age and sex, and evolving ones including virus dynamics, and increasing population immunity. Even in the presence of vaccination programmes with documented effectiveness against severe disease, ICUs should be prepared to treat patient groups with suboptimal responses to vaccination. Continued collaboration between the Nordic countries, including the harmonisation of case definitions and protocols for future studies, will improve pandemic preparedness.

**Clinical trial number:**

Not applicable.

**Supplementary Information:**

The online version contains supplementary material available at 10.1186/s12879-026-13528-8.

## Background

Since early 2020, severe acute respiratory syndrome coronavirus 2 (SARS-CoV-2) has circulated worldwide, causing a significant burden of coronavirus disease 2019 (COVID-19) on the population and healthcare systems. Some individuals with COVID-19 develop severe disease, requiring hospitalisation, and in some cases, admission to the intensive care unit (ICU). While old age and underlying medical conditions increase the risk of severe COVID-19, different variants of SARS-CoV-2 have also been associated with varying risks of severe disease [1–4]. In the beginning of the COVID-19 pandemic, healthcare systems faced substantial pressure, with some European countries reaching their ICU capacity limits [5, 6]. During the first two years of the pandemic, strict public health and social measures (PHSMs) were implemented to save lives and ensure sufficient healthcare capacity; however, measures varied between countries [7–9]. The rapid roll-out of vaccination against COVID-19 in 2021, high vaccine effectiveness against severe disease, and the global dominance of the less virulent but highly transmissible Omicron variant from late 2021 resulted in a smaller proportion of COVID-19 patients falling severely ill and in milder disease trajectories among those treated in hospitals and ICUs [2, 3, 10].

In the Nordic countries, i.e. in Denmark (population 5.9 million in 2023), Finland (5.6 million), Iceland (0.4 million), Norway (5.5 million), and Sweden (10.5 million), the first cases of COVID-19 were detected between late January and late February 2020. Following the beginning of local transmission in March 2020, PHSMs of varying strictness were implemented to limit community transmission and prevent healthcare system overload [11]. The many similarities of the Nordic countries in healthcare systems, health registries, and standard of living make them an interesting setting to study the burden of COVID-19 and the effect of such measures. In 2023, the Nordic countries initiated the Nordic follow-up project on the COVID-19 pandemic to study the burden of severe COVID-19 and different PHSMs. This study is part of the Nordic follow-up project, and here we describe and compare the acute burden of COVID-19-related ICU admissions on the population in the Nordic countries in 2020–2023 to understand the impact of the COVID-19 pandemic and inform pandemic planning.

## Methods

### Data sources and study population

All five Nordic countries have national health registries with individual-level data. Throughout the COVID-19 pandemic, the countries collected individual-level data on COVID-19-related ICU admissions using their own case definitions for ongoing surveillance by linking data from national hospital discharge registries (Denmark, Iceland) or national intensive care registries (Finland, Norway, Sweden) with data on positive tests for SARS-CoV-2 and vaccination against COVID-19. In the Nordic countries, all residents are assigned country-specific unique identifiers, which allow such registry linkages within each country. In this study, we used information on patients admitted to the ICU with confirmed COVID-19 (defined as a confirmed SARS-CoV-2 infection) in each country between 1.1.2020 and 31.12.2023. Admissions were included regardless of whether the patient was admitted directly to the ICU or to an ordinary ward first. For Denmark, Iceland, Norway, and Sweden, we included data on discharge dates, age, sex, ventilatory support use, in-ICU deaths, and primary reason for ICU admission. The primary reason for ICU admission was extracted from the intensive care registry (Sweden), the patient registry (Iceland, Denmark), or, in Norway’s case, the Norwegian Pandemic Registry. In all countries, the treating physician reported the primary reason for admission, and ICD-10 coding was used. If COVID-19 was the primary reason for ICU admission, the stay was classified as “due to COVID-19.” All patients admitted with confirmed COVID-19, regardless of the primary reason for admission, are hereinafter referred to as admitted “with COVID-19.” A detailed description of the definitions for each country is provided in Supplementary Table 1.

Information on the main reason for admission in Denmark was available from June 2020, and we categorized all patients admitted during the first half of 2020 in Denmark as having been admitted due to COVID-19. For Norway, information on the main reason for admission was available until the end of September 2023. For Finland, we used aggregated data that were publicly available during the pandemic without information on the main reason for admission. These data were available by 10-year age groups until 31.12.2022. Patients admitted to the ICU in the autonomous region of Åland (population 30,541) were included in the Finnish dataset, and additional information was obtained from a retrospective review of medical records.

Due to restrictions in sharing individual-level data internationally, each country extracted and aggregated the data for their country. Norway collated and analysed the aggregated data.

Additionally, we retrieved the monthly number of hospital admissions due to confirmed COVID-19 and population counts for each country by year and age group (Supplementary Table S1).

### Case definitions

As each country had already been collecting data on COVID-19-related ICU admissions for several years when this study was initiated, each country primarily used the case definitions routinely used in their national surveillance (Supplementary Table S1). In short, Denmark, Finland, and Sweden combined information on patients admitted to the ICU with positive test results for SARS-CoV-2, while Iceland and Norway used their hospital discharge registry or intensive care registry alone to identify patients admitted to the ICU with and due to COVID-19. In this study, data from Iceland included invasive ventilatory support only, while Denmark, Norway, and Sweden included both invasive and noninvasive ventilatory support without distinguishing between the two. These three countries defined in-ICU deaths as those occurring within 30 days after the sample date or ICU admission, while Iceland counted deaths that occurred during the hospital stay (Supplementary Table S1). All countries used a similar definition for vaccination status at admission, categorizing it as unvaccinated, vaccinated with one dose (≥21 days before admission), or vaccinated with ≥2 doses (≥7 days before admission). For patients for whom vaccination data were not available for linkage, Iceland defined the vaccination status as unknown, whereas in other countries, these patients were categorized as unvaccinated.

### Descriptive analyses

For countries with information on the main reason for admission, we focused on patients admitted to the ICU due to COVID-19. For Finland, all analyses were based on patients admitted to the ICU with COVID-19. We stratified all analyses by country and most analyses by 6-month periods, considering the dominance of different SARS-CoV-2 variants and changing vaccination coverage in the general population (Box 1; Supplementary Table S2). We calculated the 1-month incidence, 6-month-incidence by age group and the incidence for the entire study period per 100,000 inhabitants. We described the characteristics of the patients, including age (median, lower quartile, and upper quartile (LQ, UQ)), sex, and vaccination status. We also calculated the length of stay (median, LQ, UQ), and proportions of patients who received ventilatory support or died in ICU. For in-ICU deaths, we computed binomial 95% confidence intervals (CI) for the proportions (cii proportions). We also calculated the proportion of patients admitted to the ICU of all patients admitted to the hospital due to COVID-19 with binomial 95% CIs.

The analyses we done using Stata SE 18

**Box 1:** The dominance of different SARS-CoV-2 variants and the progression of the vaccination programme for COVID-19 in the Nordic countries, 2020–2023.

In all Nordic countries, different SARS-CoV-2 variants dominated for approximately one calendar year or half-year periods. The countries began their COVID-19 vaccination campaigns at the end of 2020, prioritizing nursing home residents, the elderly, and healthcare workers for vaccination. The table below shows the main dominating variant and an overview of the COVID-19 vaccination programme in the Nordic countries. A more detailed description by country is provided in Supplementary Table S2.		
**Period**	**Main dominating variant of SARS-CoV-2**	**COVID-19 vaccination programme**
Jan – Jun 2020	Wuhan	Not started
Jul – Dec 2020	Wuhan	Started in week 52 of 2020
Jan – Jun 2021	Alpha	Increasing coverage 1st dose
Jul – Dec 2021	Delta	High coverage 1st dose, increasing coverage 2nd dose
Jan 2022 – Dec 2023	Omicron	High coverage 2nd dose, subsequent doses to risk groups

## Results

During the study period, 21,587 patients were admitted to the ICU with COVID-19 in the Nordic countries, of whom approximately 50% were in Sweden (Table 1).

**Table 1:** Number of patients admitted to intensive care unit (ICU) (per 100,000 inhabitants) with COVID-19 (*N* = 21,587) and due to COVID-19 (*N* = 13,218) in the Nordic countries, 2020–2023

Country	Number of patients admitted to ICU (per 100,000)		Among patients admitted to ICU due to COVID-19	
	With COVID-19	Due to COVID-19	Received ventilatory support (%)	In-ICU deaths (%)
Denmark^1^	4,792 (81.6)	2,848 (48.5)	1,969 (69.1)	730 (25.6)
Finland^2^	2,719 (49.1)	-	-	-
Iceland	267 (68.9)	153 (39.5)	72 (47.1)	28 (18.3)
Norway^3^	3,017 (55.7)	2,036 (37.6)	1,728 (84.9)	489 (24.0)
Sweden	10,792 (103.6)	8,179 (78.5)	6,638 (81.2)	1,989 (24.3)
Total	21,587 (78.1)	13,218 (59.8)	10,407 (78.7)	3,236 (24.5)

^*1*^*Data on main reason for admission available from June 2020 onward.*^*2*^*Data on main reason for admission not available; only admissions up until 31.12.2022 are included.*^*3*^*Data on main reason for admission available up until September 2023*

### Patients admitted to the ICU with COVID-19 in Finland

**Fig. 1:**
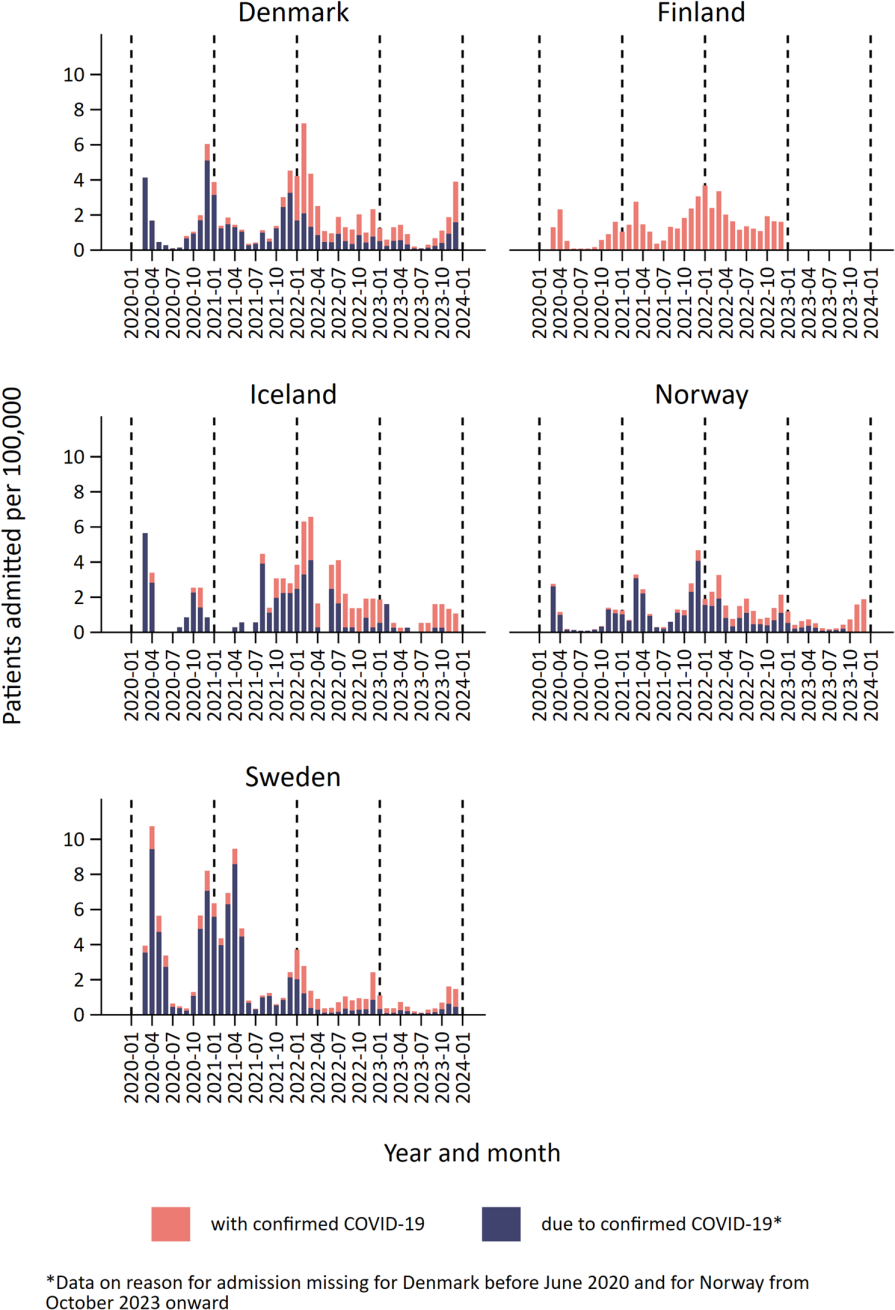
Monthly number of patients admitted to ICU with COVID-19 and due to COVID-19 per 100,000 inhabitants by country in the Nordic countries, 2020–2023

Of the 2,719 patients admitted to the ICU with COVID-19 in Finland during 2020–2022, 11 were from Åland. The incidence of patients admitted to the ICU with COVID-19 changed over time, with the highest monthly incidence registered in January 2022 (3.7 per 100,000 inhabitants) (Fig. 1).

**Fig. 2:**
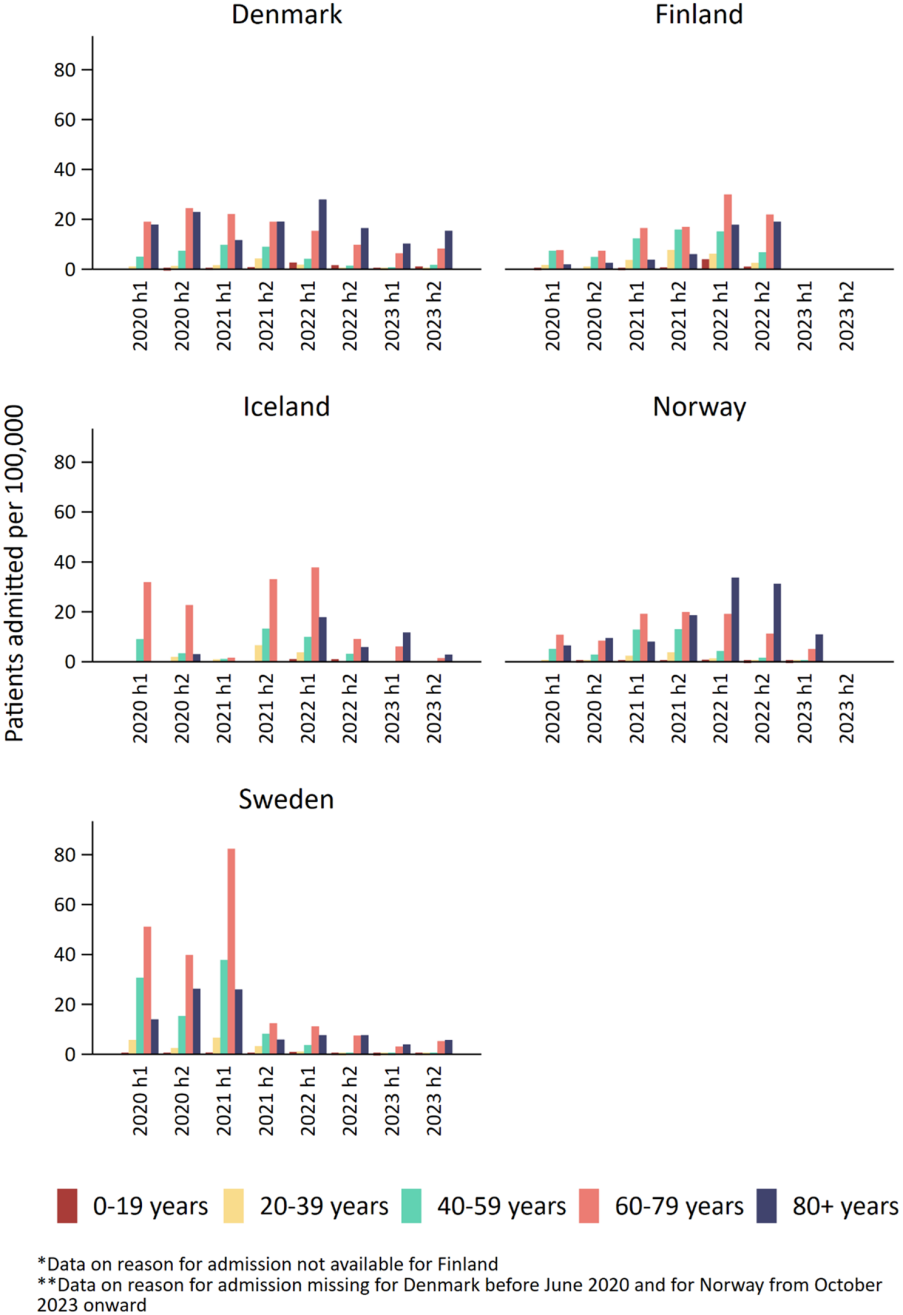
Number of patients admitted to intensive care unit with COVID-19 (finland) or due to COVID-19 (denmark, Iceland, Norway and Sweden) per 100,000 inhabitants by country, age group and 6-month period in the Nordic countries, 2020–2023

The incidence was highest in the age group 60–79 years throughout the years 2020–2022, reaching a peak 6 month-incidence of 30 per 100,000 in the first half (H1) of 2022 (Fig. 2). After a decrease in the median age in the second half (H2) of 2021, the median age increased in 2022 (Table 2, Supplementary Figure S1). The incidence among those aged under 20 years remained very low throughout 2020–2022 (Fig. 2). The percentage of patients vaccinated with ≥2 doses at admission to the ICU with COVID-19 increased from 21% in H2-2021 to 77% during H2-2022 (Table 2, Supplementary Figure S4).

**Table 2:** Characteristics of the patients admitted to ICU with COVID-19 (finland) or due to COVID-19 (denmark, Iceland, Norway and Sweden) by country and period, 2020–2023. H1: Jan–Jun, H2: Jul–Dec

Period		n	**% due to C19**^**1**^	Age			Males		Unvaccinated		Vaccinated with 1 dose		Vaccinated with ≥2 doses		Vaccination status unknown		Length of stay			Ventilatory support		In-ICU deaths	
				Median	LQ	UQ	n	%	n	%	n	%	n	%	n	%	Median	LQ	UQ	n	%	n	% (95% CI)
**Denmark**																							
H1 2020^2^		382	-	69	59	76	270	70.7	382	100.0	0	0.0	0	0.0	0	0.0	14	8	22	305	79.8	118	30.9 (26.3–35.8)
H2 2020		503	85.0	69	59	76	333	66.2	503	100.0	0	0.0	0	0.0	0	0.0	12	7	22	354	70.4	129	25.6 (21.9–29.7)
H1 2021		494	84.0	65	54	74	319	64.6	472	95.5	11	2.2	11	2.2	0	0.0	11	6	21	355	71.9	114	23.1 (19.4–27.0)
H2 2021		512	78.9	64	49	74	319	62.3	277	54.1	6	1.2	229	44.7	0	0.0	11	6	19	355	69.3	137	26.8 (23.0–30.8)
H1 2022		403	33.7	68	54	78	227	56.3	91	22.6	8	2.0	304	75.4	0	0.0	5	3	10	240	59.6	95	23.6 (19.5–28.0)
H2 2022		224	39.1	73.5	61	79	139	62.1	34	15.2	3	1.3	187	83.5	0	0.0	5	3	11	145	64.7	51	22.8 (17.4–28.8)
H1 2023		132	39.2	73	64.5	79	76	57.6	13	9.8	0	0.0	119	90.2	0	0.0	4	3	7.5	84	63.6	37	28.0 (20.6–36.5)
H2 2023		198	41.9	73	62	79	119	60.1	32	16.2	1	0.5	165	83.3	0	0.0	6	3	10	131	66.2	48	24.7 (18.9–31.4)
Total		2848	59.4	68	57	76	1802	63.3	1804	63.3	29	1.0	1015	35.6	0	0.0	9	5	17	1969	69.1	730	25.6 (24.0–27.3)
**Finland**																							
H1 2020		232	-	58	50	68	-	-	232	100.0	0	0.0	0	0.0	0	0.0	-	-	-	-	-	-	-
H2 2020		186	-	62	51	71	-	-	186	100.0	0	0.0	0	0.0	0	0.0	-	-	-	-	-	-	-
H1 2021		449	-	60	50	69	-	-	427	95.1	22	4.9	0	0.0	0	0.0	-	-	-	-	-	-	-
H2 2021		572	-	56	43	67	-	-	425	74.3	30	5.2	117	20.5	0	0.0	-	-	-	-	-	-	-
H1 2022		790	-	63	49	72	-	-	337	42.7	31	3.9	422	53.4	0	0.0	-	-	-	-	-	-	-
H2 2022		490	-	69	57	77	-	-	105	21.4	9	1.8	376	76.7	0	0.0	-	-	-	-	-	-	-
Total		2719	-	-	-	-	-	-	-	-	-	-	-	-	0	0.0	-	-	-	-	-	-	-
**Iceland**																							
H1 2020		27	93.1	64	56	67	18	66.7	27	100.0	0	0.0	0	0.0	0	0.0	7	2	18	15	55.6	4	14.8 (4.2–33.7)
H2 2020		20	80.0	69	59	70	14	70.0	19	95.0	0	0.0	0	0.0	1	5.0	6	4	21	10	50.0	3	15.0 (3.2–37.9)
H1 2021		3	100.0	58	30	69	2	66.7	2	66.7	0	0.0	0	0.0	1	33.3	6	3	10	2	66.7	1	33.3 (0.8–90.6)
H2 2021		40	76.9	60	46	69	36	90.0	14	35.0	3	7.5	17	42.5	6	15.0	7	3	13	25	62.5	6	15.0 (5.7–29.8)
H1 2022		46	56.8	66	50	78	27	58.7	13	28.3	16	34.8	14	30.4	3	6.5	3	1	8	16	34.8	10	21.7 (10.9–36.4)
H2 2022		10	23.3	71	58	76	8	80.0	0	0.0	3	30.0	6	60.0	1	10.0	4	1	7	4	40.0	2	20.0 (2.5–55.6)
H1 2023		7	50.0	79	75	84	4	57.1	2	28.6	2	28.6	3	42.9	0	0.0	2	1	6	0	0.0	1	14.3 (0.4–57.9)
	H2 2023	2	8.3	**	**	**	1	50.0	0	0.0	1	50.0	1	50.0	0	0.0	*	*	*	0	0.0	1	50.0 (1.3–98.7)
Total		153	57.3	65	52	71	110	71.9	77	50.3	25	16.3	39	25.5	12	7.8	5	2	12	72	47.1	28	18.3 (12.5–25.4)
**Norway**																							
H1 2020		208	91.6	63	53	72	157	75.5	208	100.0	0	0.0	0	0.0	0	0.0	14	7	23.5	178	85.6	37	17.8 (12.8–23.7)
H2 2020		160	89.4	64.5	55	74	115	71.9	160	100.0	0	0.0	0	0.0	0	0.0	8	4.5	17	123	76.9	36	22.5 (16.3–29.8)
H1 2021		440	90.5	59	51	69	294	66.8	434	98.6	5	1.1	1	0.2	0	0.0	10	5	19	402	91.4	74	16.8 (13.4–20.6)
H2 2021		498	84.7	60	48	72	346	69.5	327	65.7	6	1.2	165	33.1	0	0.0	10	5	20	439	88.2	102	20.5 (17.0–24.3)
H1 2022		375	61.5	70	60	78	215	57.3	106	28.3	6	1.6	263	70.1	0	0.0	4	2	11	308	82.1	113	30.1 (25.5–35.1)
H2 2022		228	51.0	76	66	81.5	144	63.2	24	10.5	1	0.4	203	89.0	0	0.0	3	1	7	174	76.3	85	37.3 (31.0–43.9)
H1 2023		94	46.1	75	67	81	58	61.7	15	16.0	1	1.1	78	83.0	0	0.0	3	2	6	77	81.9	34	36.2 (26.5–46.7)
H2 2023^3^		33	12.0	74	62	77	16	48.5	4	12.1	2	6.1	27	81.8	0	0.0	3	1	7	27	81.8	8	24.2 (11.1–42.3)
Total		2036	67.5	65	53	75	1345	66.1	1278	62.8	21	1.0	737	36.2	0	0.0	8	3	16	1728	84.9	489	24.0 (22.2–25.9)
**Sweden**																							
H1 2020		2107	86.1	61	52	69	1559	74.0	2107	100.0	0	0.0	0	0.0	0	0.0	13	6	23	1783	84.6	470	22.3 (20.5–24.1)
H2 2020		1454	84.6	66	56	74	1014	69.7	1454	100.0	0	0.0	0	0.0	0	0.0	8	3	18	1105	76.0	371	25.5 (23.3–27.8)
H1 2021		3065	90.0	63	53	72	2128	69.4	2966	96.8	75	2.4	24	0.8	0	0.0	9	4	17	2560	83.5	709	23.1 (21.6–24.7)
H2 2021		609	87.9	59	46	70	402	66.0	451	74.1	11	1.8	147	24.1	0	0.0	8	3	15	485	79.6	132	21.7 (18.5–25.2)
H1 2022		432	43.5	67	55	75	276	63.9	221	51.2	7	1.6	204	47.2	0	0.0	7	2	14	340	78.7	134	31.0 (26.7–35.6)
H2 2022		229	32.0	73	66	78	156	68.1	49	21.4	4	1.7	176	76.9	0	0.0	3	1	8	152	66.4	68	29.7 (23.9–36.1)
H1 2023		107	31.7	73	64	79	59	55.1	20	18.7	0	0.0	87	81.3	0	0.0	4	1	9	81	75.7	43	40.2 (30.8–50.1)
H2 2023		176	36.7	73	64	79	115	65.3	21	11.9	2	1.1	153	86.9	0	0.0	4	1	10.5	132	75.0	62	35.2 (28.2–42.8)
Total		8179	75.8	64	53	72	5709	69.8	7289	89.1	99	1.2	791	9.7	0	0.0	9	4	18	6638	81.2	1989	24.3 (23.4–25.3)

^*1*^*Proportion of all patients admitted to ICU with COVID-19 where COVID-19 was the main reason for admission; data for Finland not available.*^*2*^*Algorithm to identify main reason for admission implemented in June 2020 – the data for H1 2020 include all patients admitted to ICU with COVID-19.*^*3*^Information on main reason for admission available up until September 2023 **Anonymized due to small number of observations

**Fig. 3:**
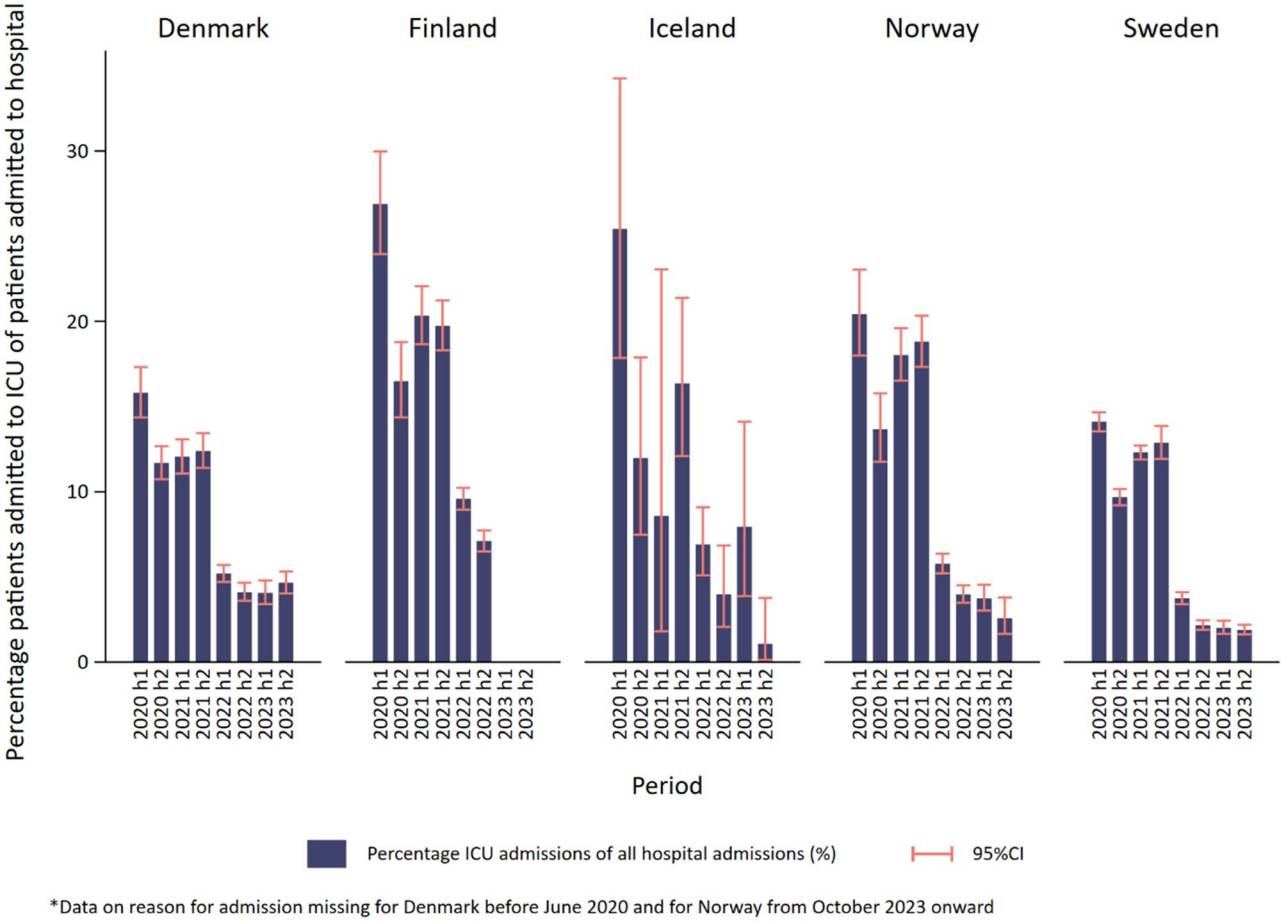
Percentage and 95% confidence intervals (CI) of patients admitted to intensive care unit (ICU) of all patients admitted to hospital with COVID-19 (finland) or due to COVID-19 (denmark, Iceland, Norway, Sweden) by country in the Nordic countries, 2020–2023

The percentage of patients admitted to the ICU of all patients admitted to the hospital with COVID-19 decreased from 27% in H1-2020 to 7% in H2-2022 (Fig. 3).

A summary of patients admitted to the ICU with COVID-19, similar to that shown in Table 2, is provided in Supplementary Table S3 for all five countries.

### Patients admitted to the ICU due to COVID-19 in Denmark, Iceland, Norway and Sweden

Of the 18,866 patients admitted to the ICU with COVID-19 in Denmark, Iceland, Norway, and Sweden, at least 13,218 (60%) were admitted due to COVID-19 (Table 1). Sweden had the highest cumulative incidence per 100,000 inhabitants (78.5, *n* = 8,179), especially during the first 1.5 years of the study period (Tables 1 and 2, Fig. 1).

Incidence varied over time, with a seemingly seasonal pattern characterized by peaks in the winter months, the highest of which occurred in March – April 2020 in Iceland and Sweden (5.6 and 9.4 per 100,000, respectively), in December 2020 in Denmark (5.1 per 100,000), and December 2021 in Norway (4.1 per 100,000).The percentage of patients admitted due to COVID-19 of all patients admitted to the ICU with COVID-19 began to decrease in early 2022 in all four countries (Fig. 1).

In 2020–2023, 62–72% of patients admitted to the ICU due to COVID-19 in each country were men (Table 2, Supplementary Figure S3). In 2020–2021, the 60–79-year-olds had the highest incidence in all four countries, with the highest 6-month incidence ranging from 20 per 100,000 in Norway to 82 per 100,000 in Sweden (Fig. 2). The median age of patients admitted to the ICU decreased in all countries in 2021 (Table 2, Supplementary Figure S2). Subsequently, the median age increased towards the end of the study period in all four countries. In Denmark and Norway, individuals aged 80 years and older had the highest incidence in 2022–2023, with the highest 6-monthly incidence reaching 28 and 34 per 100,000 inhabitants, respectively, in H1-2022 (Fig. 2). The incidence among those aged under 20 years remained very low throughout 2020–2023 in all four countries.

In Denmark, Norway, and Sweden, the percentage of patients vaccinated with ≥2 doses at admission increased from 45, 33, and 24%, respectively, in H2-2021 to 87–90% at the end of the study period (Table 2, Supplementary Figure S4). In Iceland, the percentage of patients vaccinated with ≥2 doses at admission varied between 30 and 60% from H2-2021 onward (Table 2, Supplementary Figure S4).

**Fig. 4:**
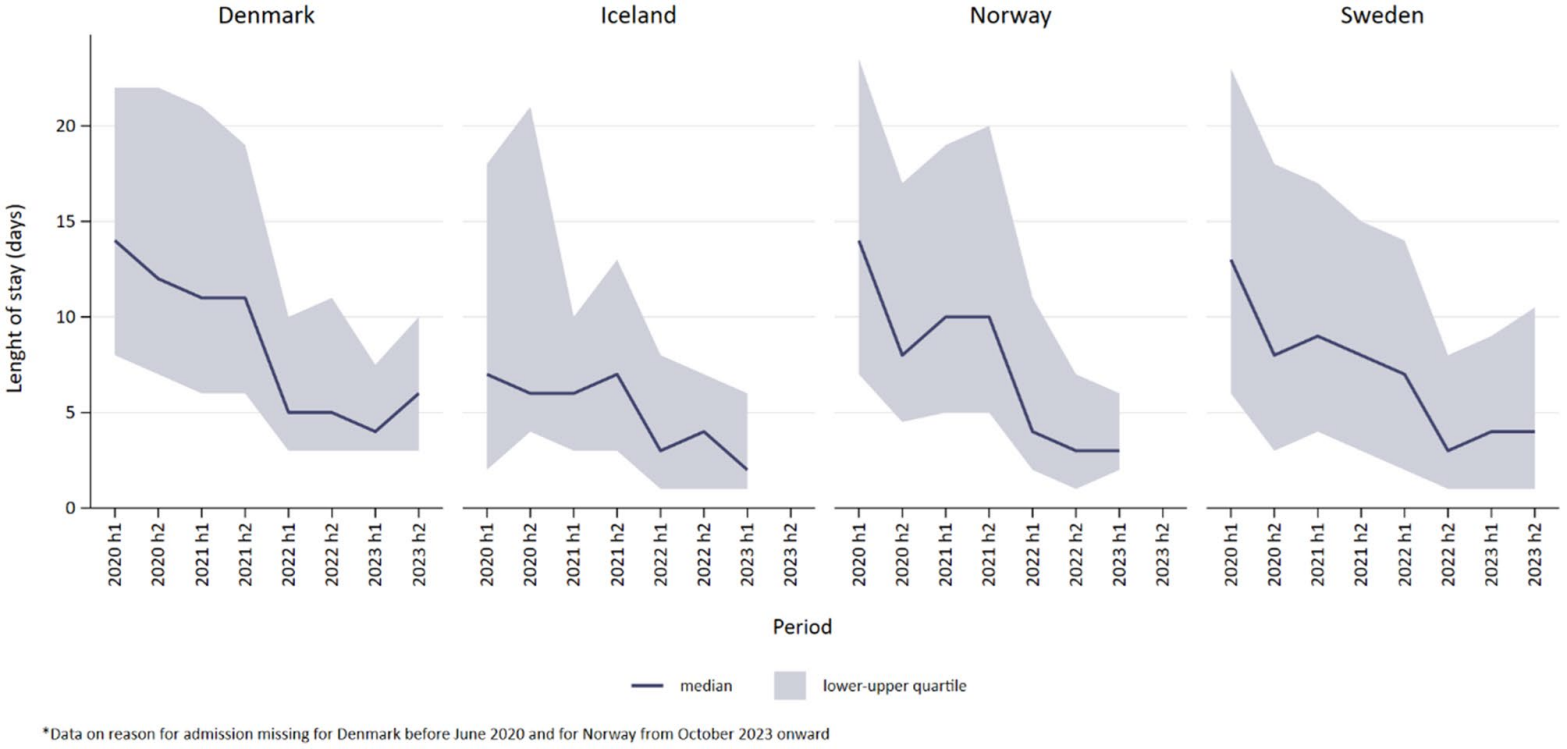
Median (lower-upper quartile) length of stay of patients admitted to intensive care unit due to COVID-19 by period and country in Denmark, Iceland, Norway and Sweden, 2020–2023

The percentage of hospitalized COVID-19 patients admitted to the ICU was highest at the beginning of the pandemic, ranging from 16% in Denmark to 25% in Iceland. It decreased over time in all four countries, reaching 2–4% (Fig. 3). The median length of stay varied between 6 and 14 days in the four countries in 2020–2021 and decreased to 2–6 days in 2022–2023 (Table 2, Fig. 4). In Denmark and Sweden, the highest percentage of patients receiving ventilatory support was observed in H1-2020 (80 and 81%, respectively), after which it decreased towards the end of the study period (Table 2, Supplementary Figure S5). In Iceland and Norway, the percentage of patients receiving ventilatory support was the highest in 2021 (63–67% and 88–91%, respectively), after which it decreased similarly to that in Denmark and Sweden. Conversely, the percentage of in-ICU deaths increased in Norway and Sweden from 18 and 22% in H1-2020, respectively, to 37 and 40% in 2022–2023 (Table 2, Supplementary Figure S6).

## Discussion

In this study, we described the acute burden of ICU admissions with and due to COVID-19 in the Nordic countries from 2020 to 2023. During this period, more than 21,000 patients were admitted to the ICU with COVID-19, of whom more than 13,000 were admitted due to COVID-19. The incidence, patient characteristics, and the severity of disease changed over time, coinciding with changes in the dominating variants of SARS-CoV-2 and increasing immunity in the population from vaccination and infection.

The incidence of ICU admissions varied in waves, with variation observed in the timing of the peak incidence across the five countries. The cumulative incidence of ICU admissions over the entire study period was highest in Sweden, reflecting more intense community transmission in 2020 and H1-2021 [12], especially in the capital region Stockholm. The reasons for the differences in transmission levels between the Nordic countries are difficult to fully assess and are outside the scope of this study. However, it was likely affected by differences in the timing and extent of the measures implemented to control the spread [11], the extent to which contact between people actually decreased, and the introduction of the virus to the respective countries [9]. A systematic review and meta-analysis estimated that PHSMs resulted in a reduction in daily case growth rates and daily ICU admissions with COVID-19 [13]. However, the effects of PHSMs should be interpreted with caution. A recent umbrella review found a lack of high-level evidence regarding the effectiveness of several commonly used non-pharmaceutical interventions [14]. From the second wave in 2021 onward, the incidence of ICU admissions was more similar between the countries. The effect of community transmission on the incidence of patients admitted to the ICU with COVID-19 was clearly observed in all countries until late 2021. In early 2022, when the Omicron variant had begun to dominate, ICU admissions due to COVID-19 declined, even though the incidence of COVID-19 reached record-high levels [12, 15]. Variation in the transmissibility and virulence of SARS-CoV-2 variants was also reflected in the percentage of patients admitted to the ICU with COVID-19 whose admission was due to COVID-19. The Omicron variant exhibited increased transmissibility yet lower virulence [16], which, together with more widespread immunity in the population, led to a lower percentage of patients being admitted to the ICU due to COVID-19.

Estimating the daily ICU bed occupancy was beyond the scope of this study. Interpreting the incidence of patients admitted due to and with COVID-19 in light of ICU capacity in the Nordic countries may provide information on the burden of COVID-19 on Nordic ICUs. In Norway and Sweden, the standard ICU capacity is 4.6 ICU beds per 100,000 inhabitants. These countries had peak incidences of 4.1 and 9.4 ICU admissions due to COVID-19 per month, respectively, with the median length of stay being approximately 10 and 14 days, respectively, at the time. This suggests a considerable burden on ICUs during the peak months, especially in Sweden. However, ICUs have a significant surge capacity, which makes it difficult to relate the monthly incidence of patients admitted to these numbers. For example, in Norway, ICUs can treat approximately three times as many patients in a crisis situation [17, 18]. Indeed, in Denmark, the number of ICU beds per 100,000 inhabitants was as high as 11.4 in H1-2020, but later it was downscaled, reaching 6.6 in H2-2023. Furthermore, comparing bed occupancy to the number of ICU beds does not consider possible challenges resulting from work absenteeism if healthcare personnel fall ill or are otherwise affected by PHSMs. In addition, assessing ICU bed occupancy at the national level may fail to detect regional differences. Stricter patient prioritization and transfers between hospitals, even internationally, can relieve pressure on ICUs with the highest burden. For example, Iceland had a preparedness plan for patient prioritization in case of capacity breaches; however, this plan was never implemented during the COVID-19 pandemic. In Åland, the threshold for ICU admission remained low throughout the pandemic.

In all countries, the majority of patients admitted to the ICU due to COVID-19 were male, and the incidence was highest among patients aged 60 years and older. While data on underlying medical risk factors was not available for our study, the findings regarding age and sex are consistent with those of previous studies [19, 20]. A meta-analysis of 59 studies found that patients aged 70 years and older were at a 2.7 times higher risk of being admitted to the ICU when infected compared to those under 70 years of age. Similarly, men had a 38% higher risk of being admitted to the ICU if infected compared to women [20]. Previous research has found that the risk of hospitalisation and ICU admission with COVID-19 varied among other population groups, with the risk of admission being higher for the foreign-born population than for the native-born population. This increased risk was likely due to socioeconomic differences, with issues such as low income, crowded living situations, and professions where it is not possible to work from home being more common in the foreign-born population [9, 21, 22]. Compared to the other Nordic countries, a larger proportion of Sweden’s population is born abroad, and a larger proportion of those born abroad are elderly [23].

During the first year of the implementation of the COVID-19 vaccination programme, the majority of patients admitted to the ICU with and due to COVID-19 were unvaccinated. In 2022–2023, the proportion of patients vaccinated with at least two doses at the time of ICU admission increased. This is expected, given the high vaccination coverage in the Nordic countries. The aim of this study was not to study the effectiveness of COVID-19 vaccines against intensive care admission. The protective effect of vaccination against severe COVID-19 has been documented in numerous studies [24]; however, some vaccinated individuals still became severely ill, as observed in our study.

The extent to which patients require invasive ventilatory support significantly affects the overall burden on ICUs. We observed that early in the pandemic, the majority of patients admitted to the ICU due to COVID-19 received ventilatory support. During this time, most ICU patients presented with severe respiratory failure, requiring ventilatory support for long periods of time [25]. Receiving ventilatory support remained common throughout the study period. In this study, we did not distinguish between invasive and noninvasive ventilatory support in the data from Denmark, Norway, and Sweden. Studies from Denmark and Norway have previously documented a decreased use of invasive ventilation from 2020 to 2022–2023 [26, 27], suggesting that noninvasive ventilation may have replaced the use of invasive ventilation to some extent during the later years of the pandemic. The decreasing length of stay and percentage of hospitalised patients requiring intensive care suggest a decrease in disease severity over time. The median length of stay was up to two weeks in some of the Nordic countries and decreased from 2022 onward, easing the burden on ICUs. Furthermore, in all countries, a smaller percentage of all patients hospitalised with and due to confirmed COVID-19 received intensive care in 2022–2023. The emergence of new, less virulent SARS-CoV-2 variants, the developments in the care of severe COVID-19, including the increased use of noninvasive ventilation, and the use of prone position and corticosteroids, and implementation of the COVID-19 vaccination programme with high vaccination coverage, especially among the elderly in all five countries, likely contributed to these changes [12, 28].

Overall, 25% of the patients admitted to the ICU due to COVID-19 in Denmark, Iceland, Norway and Sweden died. During the first half of 2020, in-ICU mortality in these countries was 23%. This proportion is significantly lower than that reported in many other European countries where ICU capacity was exceeded early in the pandemic. For instance, in Lombardy, Italy, in-ICU mortality reached 49% between February and April 2020 [29]. Although standard ICU capacity in Italy has been described as slightly higher than in e.g. Denmark, Norway and Sweden [30, 31], the cumulative incidence of reported COVID-19 cases up to early May 2020 in Italy was nearly twice as high – or more – than in most of the Nordic countries [32]. The marked strain on ICU capacity resulting from extensive community transmission likely contributed to the higher in-ICU mortality observed in Italy [33]. Our results suggest that the overall in-ICU mortality increased over time in Norway and Sweden. At the same time, the median age of the patients admitted to the ICU with and due to COVID-19 increased in all five countries. While we did not assess age group-specific mortality, a Swedish study found that despite an overall increasing in-ICU-mortality, age-group-specific survival improved among the elderly during the Omicron period [34]. Our findings together with other studies suggest that patients with suboptimal immune responses to vaccination due to old age and/or immunosuppression became over-represented among patients in ICUs in 2022–2023, often with multiorgan failure and fatal outcomes [27, 34]. At the same time, vaccination reduced the risk of severe COVID-19 in lower-risk populations [35]. Furthermore, surveillance data suggest that disease distribution in the population shifted after the removal of PHSMs in early 2022, with notable increases in SARS-CoV-2 positivity rates recorded, especially in the elderly [36]. It is unclear, however, whether the strict PHSMs implemented early in the pandemic to protect especially the oldest age groups and those with underlying medical risk factors for severe COVID-19 [37, 38] had a negative impact on these groups later in the pandemic.

An important strength of our study is that we utilised nationwide registry data used for routine surveillance of patients admitted to the ICU with and due to COVID-19 in the Nordic countries, covering the whole population in these countries. For most countries, information on the main reason for admission, laboratory test results, vaccination status and disease severity was available for the whole study period. The many similarities between the Nordic countries, including the availability of high-quality registry data, provide an interesting setting for studying potential differences between the countries. The country comparisons presented here must be interpreted with caution, considering the differences in case definitions used as well as potential differences in admission practices. In Norway, the registration of ICU admissions with COVID-19 followed the same criteria throughout the study period; however, in the other countries and regions, the case definitions were based on ICD-10 codes, the registration of which may have varied over time both between and within these countries. In Denmark, Iceland, Norway, and Sweden, we had access to individual-level data for years 2020–2023, however, only aggregated data could be shared internationally, limiting significantly the type of analyses that could be done. In Finland, we had access to only previously published, aggregated results without data on the main reason for admission for 2020–2022. This further complicated comparisons between Finland and the other countries, as focusing on patients admitted to ICU due to COVID-19 was considered methodologically sounder. However, defining the main reason for admission based on registry data can be tricky [39], and the reliability of the case definition “admitted due to COVID-19” may vary between the participating countries. Furthermore, there is currently no common definition of an ICU bed in the Nordic countries, and the patient population included in this study in each country likely included also a varying degree of patients treated in high-dependency units (HDUs). There is no international golden standard for defining an ICU admission with and/or due to COVID-19; hence, it is difficult to assess the sensitivity and specificity of the case definitions used by each country, and whether the five countries over- or underestimated the number of ICU admissions in relation to each other. Agreeing on common definitions would make such comparisons more reliable in the future. In Denmark, the algorithm used for identifying patients admitted due to COVID-19 was implemented only in June 2020. Such changes in surveillance systems may challenge also the comparison of results over time within the individual countries. Another limitation of our study is the low numbers of patients reported at times during the study period, especially in Iceland, increasing the likelihood of observing random variations instead of true changes. Furthermore, we did not specifically assess ICU bed occupancy or include data on ICU capacity in the analyses, limiting our possibilities to assess the burden on ICUs in detail.

## Conclusion and recommendations

The burden of ICU admissions with COVID-19 varied in the Nordic countries. Our findings support previous studies, showing that disease severity and therefore, the burden of ICU admissions were influenced both by non-modifiable risk factors such as high age and male sex, as well as by dynamic changes, including the intensity of community transmission, emergence of new variants of SARS-CoV-2, developments in the care of severe COVID-19, and increasing immunity in the population following vaccination, infection or both. Limiting community transmission may decrease the disease burden in the population and ICUs, especially in the beginning of a pandemic when the entire population is susceptible to the new pathogen. Even in the presence of vaccination programmes with documented effectiveness against severe disease, ICUs should be prepared to treat patient groups with suboptimal responses to vaccination. In all Nordic countries, nationwide registry data on patients admitted to ICU enabled both the ongoing surveillance of severe COVID-19 as well as this retrospective assessment of the burden of ICU admissions with COVID-19. Continued high-quality surveillance, research preparedness and further research on the implications of PHSMs for the severity of disease are needed for being better prepared for future pandemics. Close collaboration between the Nordic countries in pandemic preparedness planning, including the harmonisation of case definitions and protocols, will increase the quality of such research and future comparisons between the countries.

## Electronic supplementary material

Below is the link to the electronic supplementary material.

Supplementary material 1

Supplementary material 2

## Data Availability

The aggregated, anonymous datasets that were used to produce the tables and figures in the manuscript are available as supplementary materials. The aggregated, anonymous data used by Finland was publicly available during the pandemic. The individual-level linked data used by Denmark, Iceland, Norway and Sweden to produce the aggregated datasets cannot be shared due to legal restrictions. External researchers may apply for aggregated and/or individual-level data from the same registries as per the normal procedure for conducting health research on registry data in each country.

## Abbreviations

COVID-19: Coronavirus disease −19
H1: First half
H2: Second half
ICU: Intensive care unit
PHSM: Public health and social measures
SARS-CoV-2: Severe acute respiratory syndrome coronavirus 2
