# Supplementary material for: The burden of COVID-19-related intensive care admissions in the Nordic countries, 2020–2023
Source: BMC Infect Dis. 2026 May 19;26:1321. doi: 10.1186/s12879-026-13528-8 (PMC13366673; doi:10.1186/s12879-026-13528-8)
Supplement: Supplementary file 2 — Supplementary material 2 [file 12879_2026_13528_MOESM2_ESM.pdf]

Supplementary materials for the article: The burden of COVID-19-related intensive care admissions in the Nordic countries, 2020-2023

Table of contents

1. Definitions 2

2. The dominance of different variants of SARS-CoV-2 and the progression of the vaccination programme for COVID-19 5

3. Number of patients admitted to ICU per 100,000 by country 8

4. Characteristics of all patients admitted to ICU with confirmed COVID-19 regardless of main reason for admission 9

5. Supplementary figures based on data shown in Table 3 11

1. Definitions

Table S1. Definitions used in the analyses regarding burden of intensive care admissions with COVID-19 and due to COVID-19 in the Nordic countries, 2020-2023.

Country	Indicator	Data source(s)	Definition
**Denmark**			
	Patients admitted to hospital due to confirmed COVID-19	NPR, MiBa	Patients admitted to hospital due to covid-19 are identified by using diagnoses codes from NPR. More than 50% of the admission time must be in relation to the selected diagnosis code to count as an admission due to covid-19. Patients tested positive, an admission (not necessarily ICU) > 12 hours connected to the test
	Patients admitted to ICU with confirmed COVID-19	NPR, MiBa, The National Catalogue of Health Organisations (SOR)	Patients tested positive, an admission (not necessarily ICU) > 12 hours connected to the test, ICU admission also registered in connection to the test/admission
	Patients admitted to ICU due to confirmed COVID-19	NPR, MiBa	Patients admitted to ICU due to covid-19 are identified by using diagnoses codes from NPR. More than 50% of the admission time must be in relation to the selected diagnosis code to count as an admission due to covid-19. Patients tested positive, an admission (not necessarily ICU) > 12 hours connected to the test, ICU admission also registered in connection to test/admission.
	Time period	NPR, MiBa	Defined by the sample date of the positive test admission is connected to.
	Age	Civil Registration System (CPR)	Calculated at sample date
	Vaccination status	NPR, The Danish Vaccination Registry (DDV), MiBa	Status defined using ICU admission date and vaccination date. Three categories: Unvaccinated / only one vaccination (≥21 days before admission) / two or more vaccinations (≥7 days before admission)
	Length of stay	NPR, MiBa	Date of ICU discharge – date of ICU admission + 1
	Ventilatory support	NPR, MiBa	If one or more of the procedure code(s) for ICU admission in question starts with 'BGDA0' (Respiratory treatment) or 'BGDA1' (Non-invasive ventilation) (SKS codes – partly based on ICD10)
	In-ICU deaths	NPR, MiBa, Cause of Death Register (CoDR)	We consider death less than 30 days after sample date
	Population	CPR	Mid-year population for each year is used to estimate the population
**Finland**			
	Patients admitted to hospital with confirmed COVID-19	Care Register for Health Care (HILMO), National Infectious Disease Register (NIDR)	Patients admitted to hospital were included from HILMO if there existed a record of confirmed COVID-19 in the NIDR within a time window of -14/+7 days from the date of hospitalisation, and a relevant primary or secondary ICD-10 diagnosis code (U07.1, U07.2, J00–22, J46, J80–84, J85.1, J86). Multiple records for an individual within 30 days apart were considered as one episode.
	Patients admitted to hospital due to confirmed COVID-19	-	-
	Patients admitted to ICU with confirmed COVID-19	Finnish Intensive Care Consortium's Database	Laboratory-confirmed ICU COVID-19 admissions
	Patients admitted to ICU due to confirmed COVID-19	-	-

	Time period	Finnish Intensive Care Consortium's Database	Based on admission date
	Age	Finnish Intensive Care Consortium's Database	Defined at admission date
	Vaccination status	Finnish national vaccination register	Defined at admission date, including an assumed delay of developing protection, to as unvaccinated, vaccinated with 1 dose (≥21 days before admission) or vaccinated with >=2 doses (≥7 days before admission)
	Length of stay	-	
	Ventilatory support	-	
	In-ICU deaths	-	
	Population	Statistics Finland	The population at the end of previous year
**Iceland**			
	Patients admitted to hospital due to confirmed COVID-19	Hospital Discharge Register	All patients admitted to hospital in Iceland with a confirmed COVID-19 as defined by ICD-10 code where the main reason for admission, defined by the ICD-10 code is COVID-19
	Patients admitted to ICU with confirmed COVID-19	Hospital Discharge Register	All patients that have a registered stay in an ICU with a confirmed COVID-19 as defined by ICD-10 code (U07).
	Patients admitted to ICU due to confirmed COVID-19	Hospital Discharge Register	Same as above but the main reason for admission to hospital is COVID-19 as defined by ICD-10 code (U07)
	Time period	Hospital Discharge Register	Based on admission date
	Age	Hospital Discharge Register	Based on admission date
	Vaccination status	Vaccination Register	Defined at admission date as unvaccinated, vaccination unknown, vaccinated with 1 dose (≥21 days before admission) or vaccinated with >=2 doses (≥7 days before admission). Patients admitted to hospital that did not have an Icelandic ID number are registered as vaccination unknown as no data is available on their vaccination status
	Length of stay	Hospital Discharge Register	Defined as (last) discharge date – (first) admission date
	Ventilatory support	Hospital Discharge Register	Patients that received invasive ventilatory support. No data available on patients that received non-invasive ventilatory support
	In-ICU deaths	Hospital Discharge Register	Number of patients who died during inpatient stay that included an ICU admission
	Population	Statistics Iceland	Population for each year defined as the population at 1.1. of the year in question
**Norway**			
	Patients admitted to hospital due to confirmed COVID-19	Norwegian Pandemic Registry (NoPaR)	Patients registered in NoPaR with COVID-19 as main reason for admission. All patients admitted to hospital with confirmed COVID-19 were registered in NoPaR up to June 2023. The main reason for admission (COVID-19, other) was registered for everyone, and it was based on clinicians' assessment. Between July and September 2023, it was obligatory to register only admissions with COVID-19 as main cause. Reporting became voluntary in October 2023, and the registry was shut down shortly after. Subsequent admissions with <90 days between admission dates are considered to belong to the same infection episode
	Patients admitted to ICU with confirmed COVID-19	Norwegian Intensive Care Registry (NIR)	Patients registered in NIR as admitted with confirmed COVID-19. All patients that fulfil at least one of the following criteria are registered in NIR:

			1. Length of stay >24 hours in an intensive care unit or intensive monitoring unit, where there is a true need for intensive care or intensive monitoring<br>2. Mechanical ventilatory support<br>3. Died in an ICU<br>4. Transferred to another ICU during the first 24 hours<br>5. Continuous infusion of vasoactive medication<br>Subsequent admissions with <90 days between admission dates are considered to belong to the same infection episode
	Patients admitted to ICU due to confirmed COVID-19	NIR, NoPaR	As above, but main reason for admission (COVID-19, other) retrieved from NoPaR. Linkage based on admission and discharge dates (stays registered in NIR and NoPaR must overlap)
	Time period	NIR	Based on admission date
	Age	NIR	Defined at admission date
	Vaccination status	Norwegian Immunization Registry SYSVAK	Defined at admission date as unvaccinated, vaccinated with 1 dose (≥21 days before admission) or vaccinated with >=2 doses (≥7 days before admission). Information on vaccination was not available for patients without a permanent Norwegian ID number. These were defined as unvaccinated.
	Length of stay	NIR	Defined as (last) discharge date – (first) admission date
	Ventilatory support	NIR	Both invasive and non-invasive ventilatory support included
	In-ICU deaths	NIR	Includes deaths that occurred within 30 days after admission
	Population	Statistics Norway	Population for each year defined as the population at 1.1. of the year in question
**Sweden**			
	Patients admitted to hospital due to confirmed COVID-19	Sminet, SNPR	Patients with laboratory confirmed COVID-19 registered in Sminet that had been admitted to hospital 14 days before to 28 days after the date of COVID-19 testing, with COVID-19registered as the primary ICD-10 diagnosis in SNPR.
	Patients admitted to ICU with confirmed COVID-19	Sminet, The Swedish Intensive care Registry (SIR)	Patients with laboratory confirmed COVID-19 registered in Sminet that had been admitted to the intensive care unit regardless of primary ICD-10 diagnosis.
	Patients admitted to ICU due to confirmed COVID-19	Sminet, SIR	Laboratory confirmed covid-19 cases that had been admitted to the intensive care unit, with covid-19, viral pneumonia or acute respiratory distress syndrome registered as the primary ICD-10 diagnosis.
	Time period	SIR, SNPR	Based on admission date.
	Age	Sminet	Age at registration of covid-19 in Sminet.
	Vaccination status	The Swedish National Vaccination Register (SNVR)	Defined at admission date, including an assumed delay of developing protection, to as unvaccinated, vaccinated with 1 dose (≥21 days before admission) or vaccinated with >=2 doses (≥7 days before admission).
	Length of stay	SIR, Sminet	Defined as discharge date – admission date
	Ventilatory support	SIR	Both invasive and non-invasive ventilatory support included.
	In-ICU deaths	Sminet, SIR, the Swedish Tax Agency	Includes deaths that occurred within 30 days after admission.
	Population	Statistics Sweden	The population at the end of previous year.

2. The dominance of different variants of SARS-CoV-2 and the progression of the vaccination programme for COVID-19

Table S2. The dominance of different variants of SARS-CoV-2 and the progression of the vaccination programme for COVID-19 in the Nordic countries, 2020-2023.

Country	Period	Main dominating variant of SARS-CoV-2 (dates or weeks)	COVID-19 vaccination programme (status at the end of period unless specified otherwise)
**Denmark**			
	Jan – Jun 2020	Wuhan (from 1 Feb 2020)	NA
	Jul – Dec 2020	Wuhan (1 Jul 2020–13 Dec 2020) - Wuhan/Alpha (14 Dec 2020–31 Jan 2021)	Started week 2020-W52
	Jan – Jun 2021	Wuhan/Alpha (14 Dec 2020 – 31 Jan 2021) Alpha (1 Feb 2021–6 Jun 2021) Alpha/Delta (7 Jun 2021–9 Jul 2021)	30 Mar 2021: First dose coverage: 12.1 % - Second dose coverage: 6.4 %
	Jul – Dec 2021	Alpha/Delta (7 Jun 2021–9 Jul 2021) Delta (10 Jul 2021–14 Dec 2021) Delta/Omicron (15 Dec 2021–30 Dec 2021)	3 Oct 2021: First dose coverage: 76.02 % - Second dose coverage: 74.6 % Third dosage coverage: 1.4%
	Jan – Jun 2022	Omicron (31 Dec 2021–)	30 Mar 2022: First dose coverage: 82.3 % - Second dose coverage: 80.8 % Third dosage coverage: 61.5%
	Jul – Dec 2022	Omicron (31 Dec 2021–23 Oct 2022) Omicron/Variant-soup (24 Oct 2022–15 Jan 2023)	3 Oct 2022: First dose coverage: 81.2 % - Second dose coverage: 79.8 % Third dose coverage: 61.6% - Fourth dose coverage: 3.2%
	Jan – Jun 2023	Omicron/Variant-soup (24 Oct 2022–15 Jan 2023) Variant soup (16 Jan 2023–21 Jun 2023) EG5 variant soup (22 Jun 2023–12 Nov 2023)	21 Mar 2023: First dose coverage: 80.1 % - Second dose coverage: 78.8 % Third dose coverage: 61.1% - Fourth dose coverage: 32.0%
	Jul – Dec 2023	EG5 Variant soup (22 Jun 2023–12 Nov 2023)	Reports on vaccination coverage not generated – vaccination offered to specific groups/subpopulations
**Finland**			
	Jan – Jun 2020	Wuhan	NA
	Jul – Dec 2020	Wuhan	Started at the end of the year 2020
	Jan – Jun 2021	Alpha	Risk groups prioritized, and from older to younger age groups. First dose coverage of 80% achieved for 80+/70+ aged in March/April, for all 10-year age groups of 50+ by the end of June 2021. Second dose coverage of 80% for 80+ aged in June.

	Jul – Dec 2021	Delta	First dose coverage 70% achieved for all 10-year age groups of 20+ by the end of August. Second dose coverage of 80% for 70-79 in July, 70% for 50+ in August, and 70% for 20+ by the December 2021. Third dose coverage of 80% aged for 80+ aged in December 2021.
	Jan – Jun 2022	Omicron	Only minor increase for the first and second dose coverage. The third dose coverage above 70% for all 10-year age groups above 50 years.
	Jul – Dec 2022	Omicron	Booster doses for risk groups and oldest age groups.
	Jan – Jun 2023	Omicron	Booster doses for risk groups and oldest age groups.
	Jul – Dec 2023	Omicron	Booster doses for risk groups and oldest age groups.
**Iceland**			
	Jan – Jun 2020	Wuhan	NA
	Jul – Dec 2020	Wuhan	Started week 2020-W52
	Jan – Jun 2021	Wuhan – 28.2/Alpha 1.3 - 30.6	
	Jul – Dec 2021	Delta	Second dose coverage reached 85,9% for 18-64; 95,9% for 65+; third dose 82,9% for 65+
	Jan – Jun 2022	Omicron	Third dose for 18-64 66,5%
	Jul – Dec 2022	Omicron	Bivalent booster for 65+ 33,6%; not calculated for younger
	Jan – Jun 2023	Omicron	Not calculated
	Jul – Dec 2023	Omicron	XBB1.5 booster for 65+ 37,5%; not calculated for younger
**Norway**			
	Jan – Jun 2020	Wuhan (2020-W09 – 2021-W06)	NA
	Jul – Dec 2020	Wuhan (2020-W09 – 2021-W06)	Started week 2020-W52
	Jan – Jun 2021	Alpha (2021-W07 – 2021-W26)	Second dose coverage reached 92% among persons ≥65 years and first dose coverage reached 66% among persons ≥18 years
	Jul – Dec 2021	Delta (2021-W27 – 2021-W51)	Second dose coverage reached 89% among persons ≥ 18 years
	Jan – Jun 2022	Omicron (2021-W52 –)	Third dose coverage reached 90% among persons ≥65 years and 66% among persons ≥18 years
	Jul – Dec 2022	Omicron (2021-W52 –)	Fourth dose coverage reached 68% among persons ≥65 years and 19% among persons ≥18 years
	Jan – Jun 2023	Omicron (2021-W52 –)	Fifth dose coverage reached 13% among persons ≥65 years and 3% among persons ≥18 years
	Jul – Dec 2023	Omicron (2021-W52 –)	Booster dose coverage reached 53% among persons ≥65 years
**Sweden**			
	Jan – Jun 2020	Wuhan	NA
	Jul – Dec 2020	Wuhan	Started week 2020-W52
	Jan – Jun 2021	Alpha	Second dose coverage reached 88% among persons ≥70 years, first and second dose coverage reached 65% and 42% among persons ≥18 years, respectively
	Jul – Dec 2021	Delta	Second dose coverage reached 84% among persons ≥ 18 years

Jan – Jun 2022	Omicron	Third dose coverage reached 92% among persons ≥70 years and 66% among persons ≥18 years
Jul – Dec 2022	Omicron	Fourth dose coverage reached 81% among persons ≥65 years and 33% among persons ≥18 years
Jan – Jun 2023	Omicron	Booster dose from 2023-03-01 coverage reached 10% among persons 65-79 years, 54% among persons ≥80 years and 6% among persons ≥18 years
Jul – Dec 2023	Omicron	Booster dose from 2023-10-01 coverage reached 67% among persons ≥65 year and 12% among persons ≥18 years

3. Number of patients admitted to ICU per 100,000 by country

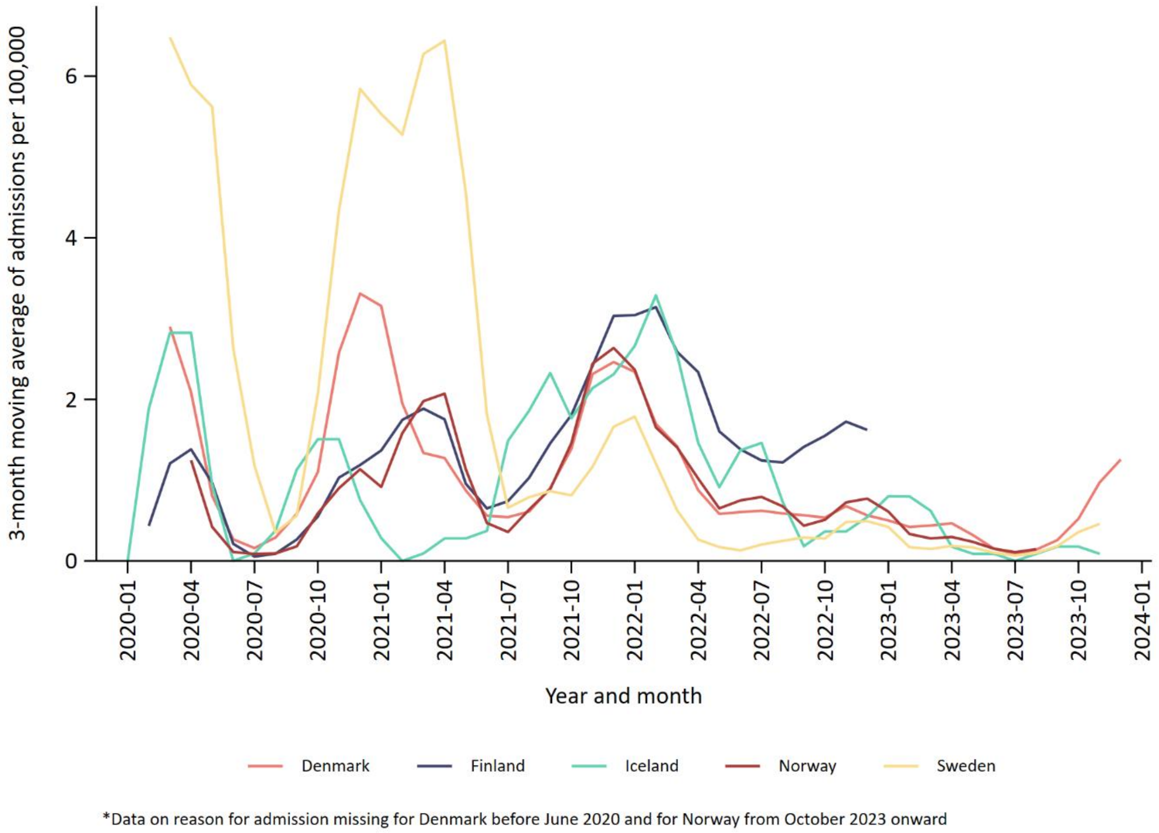

Figure S1. Three-month moving average of the number of patients admitted to intensive care unit with COVID-19 (Finland) or due to COVID-19 (Denmark, Iceland, Norway, Sweden) per 100,000 by month and country in the Nordic countries, 2020-2023

4. Characteristics of all patients admitted to ICU with confirmed COVID-19 regardless of main reason for admission

Table S3. Characteristics of the patients admitted to ICU with confirmed COVID-19 regardless of main reason for admission by country and period, 2020-2023. Note that the data for Finland are the same as presented in Table 1 in the main text. H1: Jan-Jun, H2: Jul-Dec

		Age			Males		Unvaccinated		Vaccinated with 1 dose		Vaccinated with >=2 doses		Vaccination status unknown		Length of stay			Ventilatory support		In-ICU deaths	
Period	n	Median	LQ	UQ	n	%	n	%	n	%	n	%	n	%	Median	LQ	UQ	n	%	n	%
**Denmark**																					
H1 2020	382	69	59	76	270	70.7	382	100.0	0	0.0	0	0.0	0	0.0	14	8	22	305	79.8	118	30.9
H2 2020	592	69	58	76	390	65.9	592	100.0	0	0.0	0	0.0	0	0.0	11	6	21	399	67.4	153	25.8
H1 2021	588	65	54	74	370	62.9	558	94.9	17	2.9	13	2.2	0	0.0	10	5	19	395	67.2	135	23.0
H2 2021	649	63	47	74	397	61.2	332	51.2	9	1.4	308	47.5	0	0.0	9	5	17	411	63.3	162	25.0
H1 2022	1197	65	46	77	694	58.0	227	19.0	17	1.4	953	79.6	0	0.0	4	2	7	524	43.8	228	19.0
H2 2022	573	71	56	78	330	57.6	71	12.4	6	1.0	496	86.6	0	0.0	5	3	9	303	52.9	143	25.0
H1 2023	337	72	61	78	195	57.9	36	10.7	2	0.6	299	88.7	0	0.0	4	3	9	189	56.1	88	26.1
H2 2023	472	68	53.5	78	275	58.3	95	20.1	5	1.1	372	78.8	0	0.0	5	3	9	243	51.5	102	21.6
Total	4790	67	54	76	2921	61.0	2293	47.9	56	1.2	2441	51.0	0	0.0	6	3	14	2769	57.8	1129	23.6
**Finland**																					
H1 2020	232	58	50	68	-	-	232	100.0	0	0.0	0	0.0	0	0.0	-	-	-	-	-	-	-
H2 2020	186	62	51	71	-	-	186	100.0	0	0.0	0	0.0	0	0.0	-	-	-	-	-	-	-
H1 2021	449	60	50	69	-	-	427	95.1	22	4.9	0	0.0	0	0.0	-	-	-	-	-	-	-
H2 2021	572	56	43	67	-	-	425	74.3	30	5.2	117	20.5	0	0.0	-	-	-	-	-	-	-
H1 2022	790	63	49	72	-	-	337	42.7	31	3.9	422	53.4	0	0.0	-	-	-	-	-	-	-
H2 2022	490	69	57	77	-	-	105	21.4	9	1.8	376	76.7	0	0.0	-	-	-	-	-	-	-
Total	2719	-	-	-	-	-	-	-	-	-	-	-	0	0.0	-	-	-	-	-	-	-
**Iceland**																					
H1 2020	29	64	55	68	19	65.5	29	100.0	0	0.0	0	0.0	0	0.0	7	2	18	16	55.2	4	13.8
H2 2020	25	69	59	70	19	76.0	24	96.0	0	0.0	0	0.0	1	4.0	6	3	24	11	44.0	3	12.0
H1 2021	3	58	30	69	2	66.7	2	66.7	0	0.0	0	0.0	1	33.3	2	1	8	2	66.7	1	33.3
H2 2021	52	61	42	70	45	86.5	20	38.5	6	11.5	20	38.5	6	11.5	3	1	6	31	59.6	8	15.4
H1 2022	81	65	49	74	51	63.0	18	22.2	30	37.0	28	34.6	5	6.2	3	1	8	26	32.1	19	23.5
H2 2022	43	67	58	76	34	79.1	7	16.3	12	27.9	16	37.2	8	18.6	6	1	7	9	20.9	11	25.6
H1 2023	14	75	65	84	9	64.3	3	21.4	5	35.7	6	42.9	0	0.0	3	2	8	3	21.4	3	21.4
H2 2023	24	71	59	77	13	54.2	4	16.7	9	37.5	8	33.3	3	12.5	3	1	6	0	0.0	3	12.5
Total	267	67	55	73	192	71.9	107	40.1	62	23.2	74	27.7	24	9.0	4	2	10	98	36.7	52	19.5

**Norway**																					
H1 2020	227	63	53	72	168	74.0	227	100.0	0	0.0	0	0.0	0	0.0	14	7	23	190	83.7	42	18.5
H2 2020	179	65	54	75	128	71.5	179	100.0	0	0.0	0	0.0	0	0.0	8	4	16	137	76.5	42	23.5
H1 2021	486	59	51	69	325	66.9	477	98.1	5	1.0	4	0.8	0	0.0	10	5	18	437	89.9	83	17.1
H2 2021	588	60	47.5	72	411	69.9	380	64.6	9	1.5	199	33.8	0	0.0	9	4	19	515	87.6	127	21.6
H1 2022	610	70	58	78	370	60.7	154	25.2	13	2.1	443	72.6	0	0.0	3	1	9	461	75.6	183	30.0
H2 2022	447	74	65	80	283	63.3	58	13.0	7	1.6	382	85.5	0	0.0	3	1	7	316	70.7	154	34.5
H1 2023	204	75	65.5	80	125	61.3	34	16.7	2	1.0	168	82.4	0	0.0	3	1	6	152	74.5	79	38.7
H2 2023	276	74	63	79	156	56.5	42	15.2	3	1.1	231	83.7	0	0.0	2	1	6	199	72.1	80	29.0
Total	3017	67	54	76	1966	65.2	1551	51.4	39	1.3	1427	47.3	0	0.0	6	2	13	2407	79.8	790	26.2
**Sweden**																					
H1 2020	2447	61	52	69	1794	73.3	2447	100.0	0	0.0	0	0.0	0	0.0	12	5	22	1976	80.8	557	22.8
H2 2020	1718	66	56	74	1183	68.9	1718	100.0	0	0.0	0	0.0	0	0.0	7	3	16	1251	72.8	433	25.2
H1 2021	3407	63	53	72	2349	68.9	3285	96.4	93	2.7	29	0.9	0	0.0	8	3	16	2738	80.4	788	23.1
H2 2021	693	58	45	70	445	64.2	504	72.7	13	1.9	176	25.4	0	0.0	7	3	14	534	77.1	143	20.6
H1 2022	994	65	46	74	598	60.2	379	38.1	25	2.5	590	59.4	0	0.0	3	1	8	633	63.7	257	25.9
H2 2022	716	71	58	78	453	63.3	148	20.7	12	1.7	556	77.7	0	0.0	2	1	5	406	56.7	176	24.6
H1 2023	338	71	60	78	199	58.9	58	17.2	5	1.5	275	81.4	0	0.0	2	1	5	213	63.0	101	29.9
H2 2023	479	72	62	79	293	61.2	61	12.7	6	1.3	412	86.0	0	0.0	3	1	7	313	65.3	142	29.6
Total	10792	64	53	73	7314	67.8	8600	79.7	154	1.4	2038	18.9	0	0.0	7	2	15	8064	74.7	2597	24.1

5. Supplementary figures based on data shown in Table 3

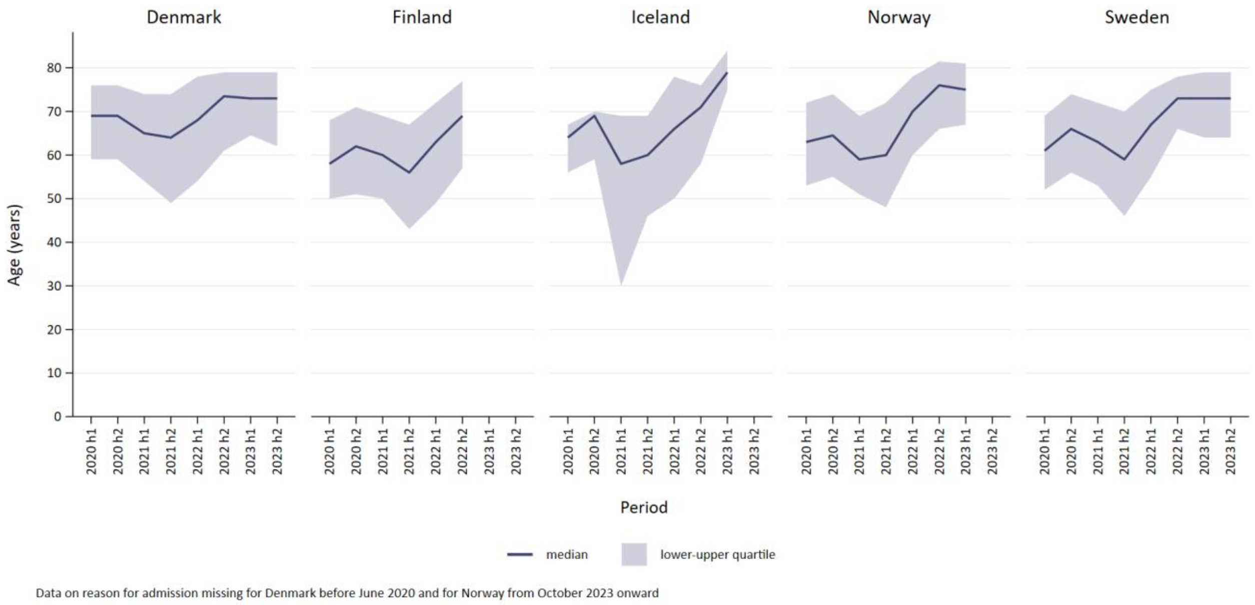

Figure S2. Median (lower-upper quartile) age of patients admitted to ICU due to COVID-19 by period and country in the Nordic countries, 2020-2023

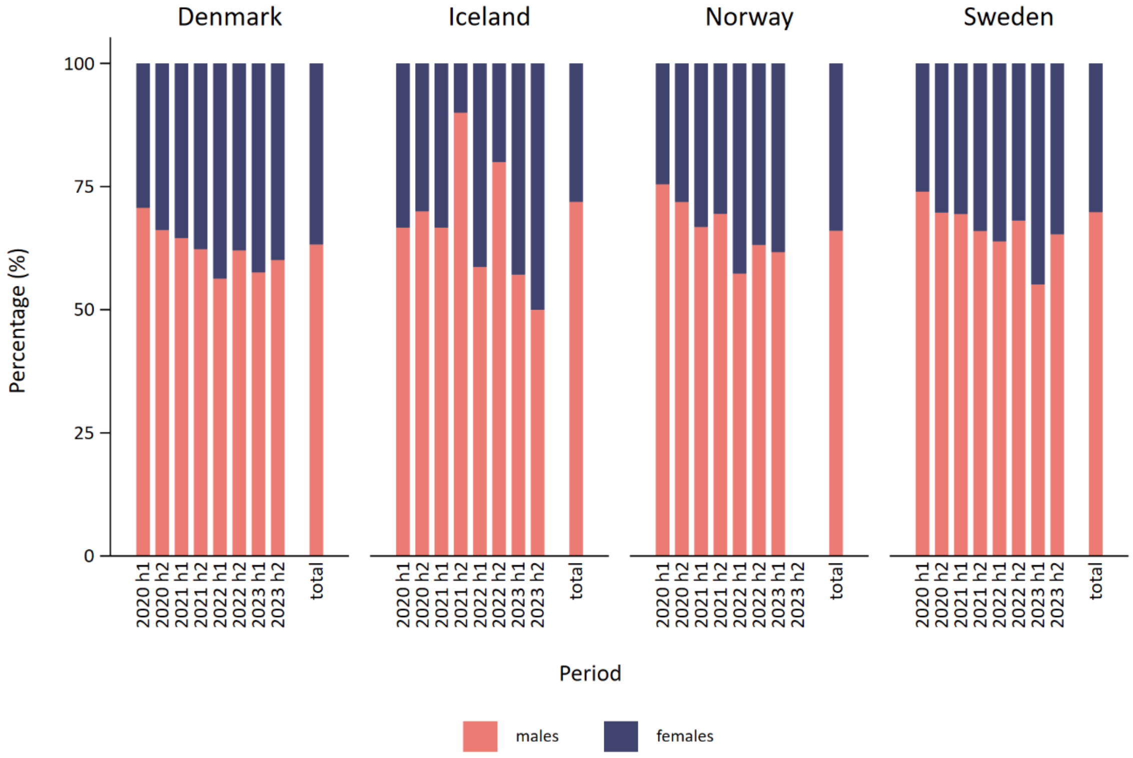

*Data on reason for admission missing for Denmark before June 2020 and for Norway from October 2023 onward

Figure S3. Sex distribution among patients admitted to intensive care unit due to confirmed COVID-19 by period and country, 2020-2023

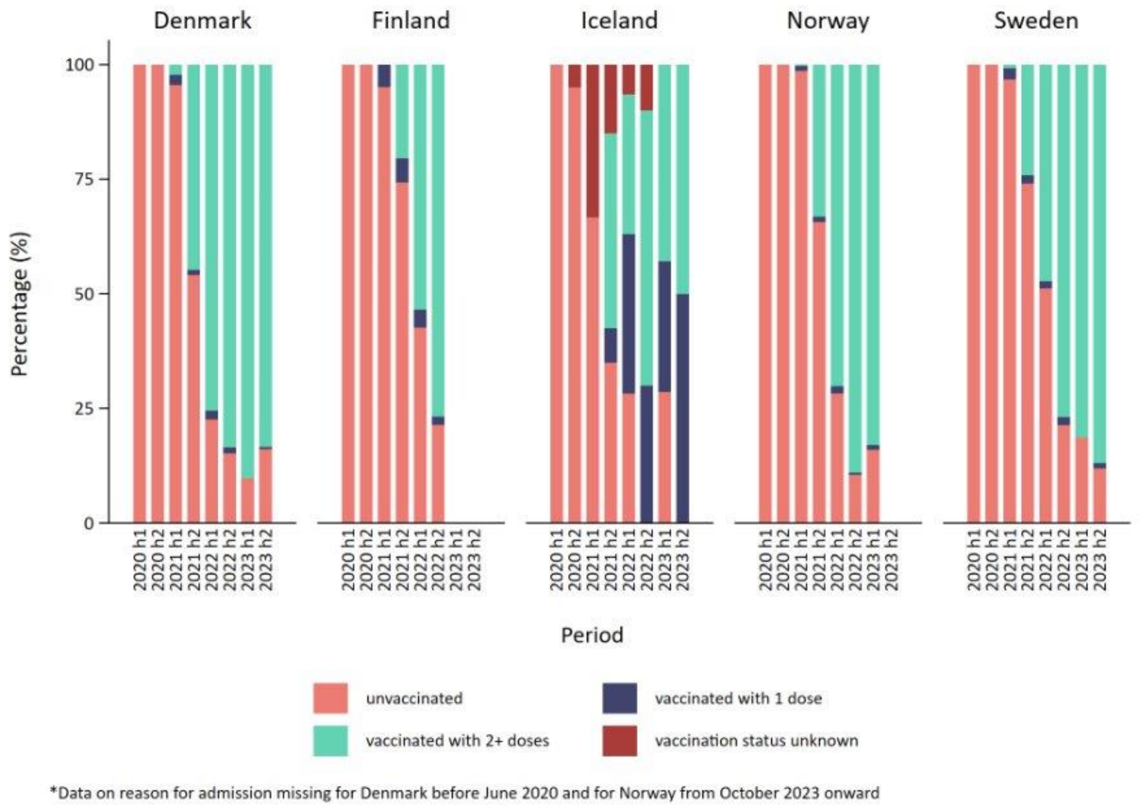

Figure S4. Vaccination status among patients admitted to intensive care unit due to confirmed COVID-19 by period and country in Denmark, Iceland, Norway and Sweden, 2020-2023

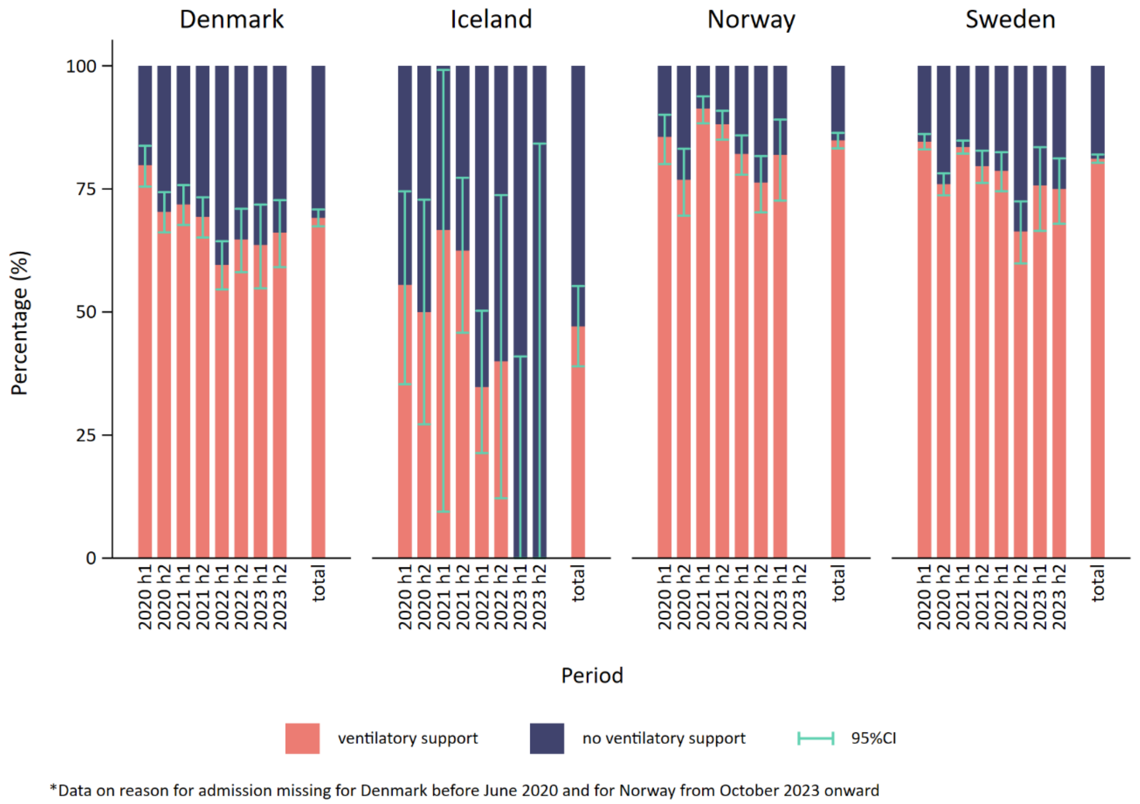

Figure S5. Percentage of patients admitted to intensive care unit due to COVID-19 who received ventilatory support by period and country in Denmark, Iceland, Norway and Sweden, 2020-2023

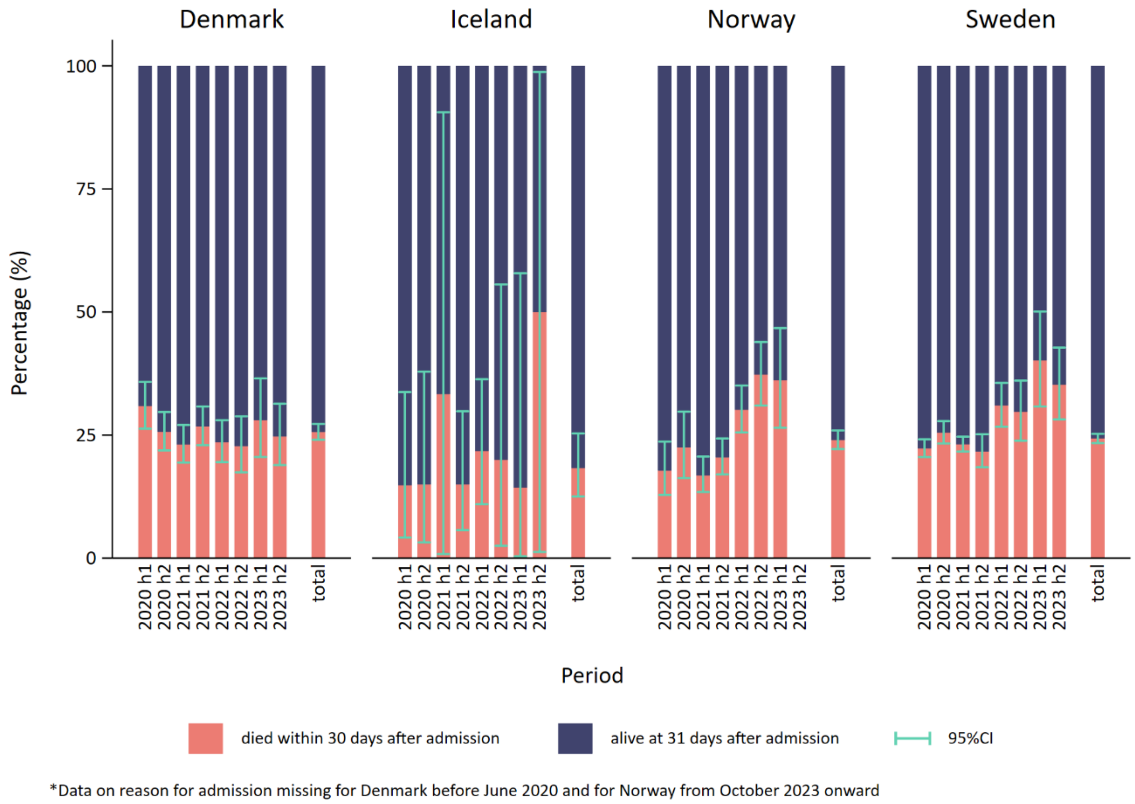

Figure S6. Percentage of in-ICU deaths among patients admitted to intensive care unit due to COVID-19 by period and country in Denmark, Iceland, Norway and Sweden, 2020-2023
